# Evaluating the implementation of a patient engagement mHealth application in clinical infection prevention

**DOI:** 10.1017/ice.2025.10211

**Published:** 2025-10

**Authors:** Robbert Gerard Bentvelsen, Rosalie van der Vaart, Niels H. Chavannes, Karin Ellen Veldkamp

**Affiliations:** 1 LUCID Medical Microbiology and Infection Prevention, Leiden University Medical Centre, Leiden, The Netherlands; 2 Microvida Laboratory for Microbiology, Amphia Ziekenhuis, Breda, The Netherlands; 3 Unit of Health, Medical and Neuropsychology, Faculty of Social and Behavioral Sciences, Leiden University, Leiden, The Netherlands; 4 Centre of Expertise Health Innovation, The Hague University of Applied Sciences, The Hague, The Netherlands; 5 Public Health and Primary Care, National eHealth Living Lab, Leiden University Medical Centre, Leiden, The Netherlands

**Keywords:** Nosocomial infection, Implementation Research, CAUTI, Patient Engagement, mHealth, CFIR

## Abstract

**Objective:**

Successful implementation of patient engagement (PE) and mHealth could reduce inappropriate catheter use and Catheter-associated urinary tract infections (CAUTIs). Insight into patient acceptance, impact on PE and quality of care, potential barriers and facilitators to the implementation of an mHealth intervention could improve the impact of both current and future infection prevention programs.

**Methods:**

Implementation of the smartphone app “Participatient” was evaluated in four Dutch hospitals. Patient questionnaires assessed the acceptability of the app and its impact. Healthcare professionals (HCPs) were interviewed to evaluate the implementation process.

**Results:**

Acceptability constructs were evaluated positively. PE and quality of care were rated high before and after implementation. All 22 HCPs perceived barriers, eg incomplete training for HCPs and unclear communication on roles; and lack of promotion by ward professionals. The principal facilitator was the HCPs’ positive attitude toward PE.

**Conclusions:**

App users perceived the Participatient app as acceptable, which fulfills a precondition for implementation. The implementation strategy evaluated in the present study was designed to fulfill all the conditions considered crucial for implementation. Nevertheless, the level of adoption remained low, and HCPs still imputed their failure to promote the use of the app to insufficiencies in training and communication and to a misfit between the app and their existing workflow. These findings underscore the need to verify whether there may be additional, less evident barriers to the adoption of mHealth tools that support PE in general, and more specifically, to the adoption of Participatient to engage patients in preventing CAUTIs.

## Introduction

Catheter-associated urinary tract infections (CAUTIs) are a main cause of healthcare-associated infections that lead to an increased burden of disease, increased use of antibiotics, and prolonged hospital stays.^1^ Most previously reported strategies to prevent CAUTIs have attempted to increase healthcare professionals’ (HCPs) awareness of inappropriate placement and prolonged catheter use via education, increased surveillance with feedback, and reminders for timely removal.^2–4^ These interventions have shown varying degrees of success.

To date, active patient involvement in decision-making regarding catheter use has been very limited.^5^ Patient engagement (PE) is in line with the transition to shared decision-making in healthcare.^6^ Patients prefer to be involved in their treatment and tend to prefer a less invasive treatment option.^7^ Hence, patient engagement may reduce overuse of medical devices such as catheters and thereby reduce infections. PE increases patients’ feeling of being informed and satisfied with the decision-making process.^8^ Patients could be more involved in their treatment process by providing plain language information and easy-to-use monitoring tools. Through dialog with their HCPs, patients can play a role in signaling and preventing infection risks, such as unnecessarily prolonged catheter use.

Digital applications (apps) could facilitate PE since apps could help to inform and empower patients. Various medical and public health practices supported by mobile devices (mHealth) have been developed to involve patients in their healthcare and in infection prevention.^9^

The goal of the intervention was to create awareness for inappropriate catheter use through stimulating PE by introduction of the Participatient app.^10^ Within the app, patients are motivated to check their catheter indication and to discuss this indication with HCPs. The implementation was part of the PECCA trial (Patient Engagement Counter CAUTI with an App). Details and results on the development of the app’s content and on its efficacy in reducing inappropriate catheter use have been previously published.^10–12^

In this study, we evaluated the implementation of the Participatient app. We aimed to assess the acceptability of the app as a precondition for successful implementation, and to determine whether the intervention impacts PE and perceived quality of care. The main aim of this implementation study was to assess relevant barriers and facilitators to the implementation of the intervention.

## Methods

### Design

This is a multicenter mixed-method implementation study. We assessed the acceptability of the app; the impact of the intervention; and the barriers and facilitators for implementation. The intervention was implemented in four acute care hospitals in the Netherlands.

**Figure 1.**
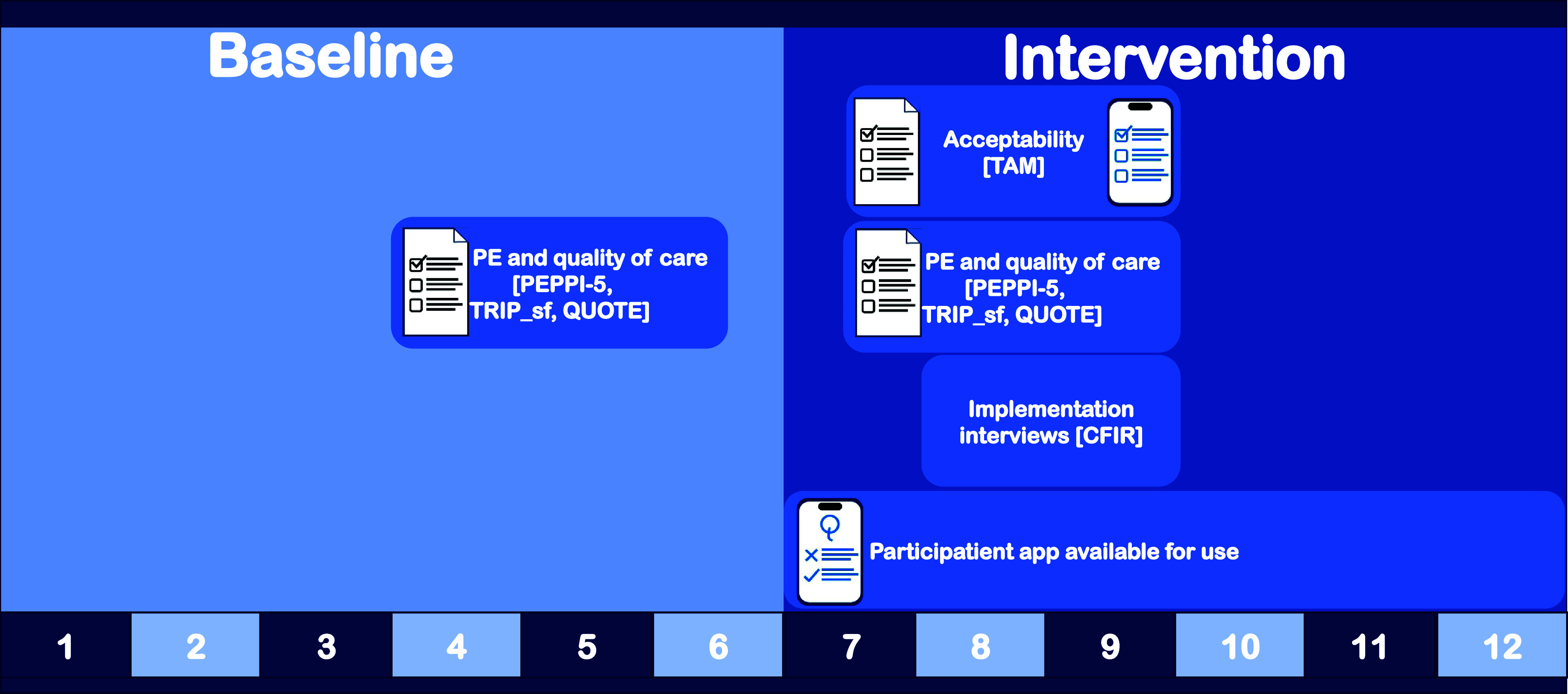
Timeline of the implementation evaluation of the mHealth intervention. Timeline comprising the six-month baseline and six-month intervention periods. The current study comprises an evaluation of the implementation of the mHealth intervention. The data were collected through a pen-and-paper questionnaire on patient engagement (PE), quality of care, and acceptability; an in-app questionnaire on acceptability; and implementation interviews. The instruments and framework used are indicated between square brackets and described in detail in the supplement (S4). Questions on quality of care were based on the “perceived efficacy in patient–physician interactions” using the PEPPI-5 questionnaire; “trust in physicians” using the TRIP_sf questionnaire and the “quality of care through the patient’s eyes” of the physician and nurse using the QUOTE. Acceptability was assessed through the Technology Acceptance Model (TAM). Interviews were conducted using the Consolidated Framework for Implementation Research (CFIR).

To assess the impact of the intervention on PE and quality of care, data were collected three months before implementation and three months after implementation (Figure 1). After implementation, questions concerning the acceptability of the app were included in a paper questionnaire (Supplement S3). Additionally, these acceptability questions were part of a digital questionnaire within the app. App users receive prompts with a request to evaluate their use of the app. By combining the pen-and-paper responses with the digital in-app responses, we sought to sample a representative patient population, including those who opted not to use the app.

All patients present at the wards on the days of the survey were asked to complete paper questionnaires. Patients were excluded if they were younger than 18 years, unable to read Dutch, cared for by an HCP at the time of the survey, or admitted to the hospital on the day of the survey. Due to privacy concerns, no unique identifiable data were asked.

The barriers to and facilitators of implementation were assessed through semi-structured interviews with HCPs. The interviews took place on the wards one month after the implementation of Participatient.

The research team consisted of two psychologists in training and a physician-scientist, supervised by senior experts. This study was approved by the Medical Ethical Committee (MEC) Leiden and registered (NL7178). Considering the original protocol,^13^ the results concerning urinary catheter use and infections were previously published.^10^

### mHealth intervention

With the patient-centered app, patients can monitor their catheter indications and are encouraged to engage with their HCPs.^11^ As part of the implementation, clinical lessons were provided on each ward. The lessons for HCPs comprised PE, the app and its contents, the implementation plan on the wards, and the HCPs roles in supporting patients in installing and using the app. To reach as many HCPs as possible, four lessons per ward were offered encouraging interaction and discussion. HCPs were asked for suggestions for additional app features, and they were invited to become their ward’s very important participant (VIP). A VIP would be more involved in the implementation process as a liaison between the ward and the research team to create a base of support for the project.

After preparation through clinical lessons, an educational kick-off day was organized at each ward to mark the start for patients. Patients were enabled to download and use the Participatient app. The app was introduced to patients through a leaflet for installation handed out by HCPs at admission. Alternatively, informal caregivers such as family and friends could use the app for incapable patients.

After the kick-off day, the HCPs were reminded of the project via posters on the wards and periodic emails. In addition, the research team offered technical support for patients and HCPs in fortnightly support rounds.

### Assessment instruments

The acceptability of the app was assessed with the Technology Acceptance Model (TAM)^14^ to develop a questionnaire (Tables 1 and S3).

**Table 1.** Acceptability of the participatient app

Measurement scale	Statement	N	Score (SD; range)	n (%)
Ease of use	Using the app is easy	55	4.0 (0.79; 2–5)	
Ease of use	It is easy to find the information I need in the app	54	4.0 (0.73; 2–5)	
Ease of use	The information in the app is easy to understand	52	4.1 (0.69; 2–5)	
Usefulness	I find the information in the app useful	51	3.6 (0.85; 2–5)	
Usefulness	Due to this app, I feel more involved in my own healthcare	50	3.4 (0.76; 2–5)	
Usefulness	I find it useful to receive advice to discuss with my doctor/nurse	55	3.7 (0.88; 1–5)	
Future use	During a following hospitalization, I will use the app again	49	2.5 (0.65; 1–3)	
	Yes			28 (57%)
	Maybe			17 (35%)
	No			4 (8%)

The acceptability questions using the Technology Acceptance Model (TAM) consisted of three items on perceived usefulness, three items on perceived ease of use and one item on intention for future use. The items were formulated as statements, and the participants could choose from responses on a Likert scale ranging from 1 (strongly disagree) to 5 (strongly agree). The scores for the intention to future use ranged from 1 (no), 2 (may) to 3 (yes). The responses were valid when at least three items were included.

The assessment of PE and of the quality of care was operationalized via validated measures of “perceived efficacy in patient–physician interactions,”^15,16^ “trust in physicians,”^17^ and “quality of care through the patient’s eyes” of both the physician and the nurse.^18,19^ One question was added to measure overall perceived quality of care (Table S4).

To assess the facilitators and barriers throughout the implementation, the consolidated framework for implementation research (CFIR) was used.^20^ The CFIR is a framework designed to guide formative evaluation and considers five implementation domains. The CFIR domains were used to guide semi-structured HCP interviews that focused on identifying the barriers and facilitators to clinical implementation. The responses were grouped per theme and organized per domain.

### Data analyses

Data were analyzed using SPSS. The acceptability subscales “ease of use” and “usefulness” were analyzed via descriptive statistics. In the PE and quality-of-care analyses, the internal consistency between scores within each scale was calculated with Cronbach’s alpha. The equality of variance between groups at baseline and during the intervention was assessed using Levene’s test. Differences were considered significant if the *P*-value was <0.05. For the interviews, themes were generated using the framework method.^21^

## Results

In total, 249 users of the Participatient app consented to share user data. Per week, a median of 7 (IQR: 5–13) new users registered. Details on user characteristics were discussed in our previous paper.^10^

### Acceptability

Table 1 lists the outcomes of the analysis of the 55 responses on the acceptability of the app. The ease-of-use rating was 4.1 (SD 1.7) out of five, and the usefulness rating 3.6 (SD 1.9). Four of the app users (8%), had no intention to use the app during a subsequent hospitalization, 57% (28) did intend future use, and 35% (17) would consider it.

### PE and quality of care

Paper questionnaires regarding PE and quality of care were completed before and after the intervention by 458 patients. The sociodemographics of the control group were comparable to those of the intervention group (Table S1).

Table 2 presents the results of the measures of perceived efficacy in patient–physician interactions; trust in their physician; the perceived quality of care of their physician and nurse, and the perceived overall quality of care. All the constructs had sufficient reliability within the scales. Scores on all scales were high, with quality of care at baseline 8.2 and after the intervention 8.5. The scores for trust in their physician were significantly higher after the intervention. No other changes were found between baseline and after the intervention.

**Table 2.** Patient Engagement and Quality of Care scores at baseline and after implementation of the mHealth intervention

		*Baseline*						*Intervention*						
Subscale	Items	N	Mean	SD	Min	Max	α	N	Mean	SD	Min	Max	α	*P*-value
Efficacy in interactions	5	249	4.12	0.51	1	5	0.85	166	4.14	0.46	3	5	0.83	0.69
Trust in their physician	3	250	3.63	0.54	1	4	0.91	173	3.74	0.43	2	4	0.90	0.03
Quality of physician	6	244	3.51	0.44	1	4	0.85	162	3.57	0.45	1	4	0.88	0.21
Quality of nurse	6	246	3.52	0.45	1	4	0.86	158	3.50	0.47	1	4	0.91	0.80
Quality of care overall	1	268	8.2	1.21	2	10	n.a.	190	8.5	1.05	5	10	n.a.	0.49

The possible range for the five questions on “perceived efficacy in patient–physician interactions” using the PEPPI-5 questionnaire is 1 (no trust) to 5 (a lot of trust); for three questions on “trust in physicians” using the TRIP_sf questionnaire and the “quality of care through the patient’s eyes” of the physician and nurse using the QUOTE, the range is 1 (poor quality) to 4 (optimal quality). The overall perceived quality of care of the hospital ranged from 1 (very bad quality) to 10 (very good quality). SD = standard deviation, Min and Max represent the range of scores, and α represents Cronbach’s alpha for internal consistency in the case of multiple questions per construct. All the constructs had a Cronbach’s alpha above 0.80, indicating sufficient reliability within the scales.

### Implementation evaluation

A total of 22 HCPs from four hospitals were interviewed (Table S2). Twenty of the interviewees (91%) were female. Eleven interviewees were nurses (50%), eight were senior nurses (36%), and three were team managers (14%). The facilitators and barriers perceived during the implementation of the mHealth intervention are summarized in Table 3 and described below per CFIR domain.

**Table 3.** Barriers and Facilitators for implementation of the mHealth intervention per the CFIR implementation domain

Implementation domain	Theme	Example quotes	Mentioned
Intervention characteristics	Fit of app components with the needs of the hospital ward	The catheter check and the pain score could be useful, even though there is already a main focus on them on our ward. I find the information about the ward the most useful (PP18).	Often
	Perceived effect of the app	The catheter check makes the patients aware that they could participate and engage in their own healthcare process (PP4).	Sometimes
	User-friendliness	I find it very easy to use. Easy to navigate in (PP11).	Sometimes
	Fit with digitalization within hospitals	Currently, I find an app very useful. Apps are used a lot and almost everyone has a smartphone (PP16).	Sporadic
Inner setting	Promotion by HCPs within hospital ward	I have not noticed any results of the app. I have never seen a patient who downloaded the app (PP12).	Often
	Investment of HCPs	Certain activities have less priority than the primary healthcare process (PP13).	Often
	Suitability of the patient population	We have a mixed patient population. 18–108 years, many nationalities, non-Dutch patients. The app is not relevant for everyone (PP10).	Often
	Attitude toward innovations	Our team encourages innovation and improvements in healthcare. I’m sure they also encouraged a new app (PP9).	Sometimes
	Embedding within current working method	The app is not part of our routine. That’s the biggest problem, as it is not difficult to promote (PP17).	Sometimes
	Stability within the team	We tried to inform as many nurses as possible, however, last year, there have been enormous changes within the team (PP22).	Sporadic
Outer setting	Reorganization within hospital	There has been some commotion due to changes in the hospital organization. Unfortunately, we could not foresee that (PP16).	Sporadic
	Vision on shared decision-making and patient engagement	The hospital is very eager to implement shared decision-making, and more and more information is provided to the patient to be able to help decide themselves what is and is not good for them (PP18).	Sporadic
Characteristics of individuals	Attitude toward patient engagement	Engaging patients in their own healthcare fits with the present time. I even think that patients want to be involved. People are more outspoken and are able to search everything on the internet. In the past, people thought that whatever a doctor said, it was the truth. Currently, people feel more independent and responsible (PP20).	Often
	Attitude toward the app	The app is very convenient. I’m most enthusiastic about the information provision for patients (PP16).	Sometimes
	Characteristics of patients	I think the interest of a patient depends on the characteristics of a patient. Some patients want to know everything about their healthcare situation, while others do not. It is very personal (PP5).	Sometimes
	Organization of implementation events	I think that the main problem is that not everyone has followed the clinical lessons and that the lessons did not fully address the added value of the app (PP1).	Often
Implementation process	Clarity about goal of the app, responsibility, expectations and roles	I don’t think that our role in the implementation process has been clearly communicated (PP1).	Often
	Involvement in the process	If we would have taken more responsibility and if we would have adjusted the content of the app with the app developers, it might have been better for the implementation (PP11).	Often
	Promotion material	I have seen the posters and banner; they were quite clear. However, we do not want too many posters all around the ward, so they have been removed (PP10).	Sometimes
	Follow-up of the implementation	Maybe Participatient could be emphasized by organizing a “Participatient-week.” If you do so, it will be remembered by the nurses (PP17).	Sometimes

Overview of the coded interview responses from healthcare professionals (HCPs) with relevant example quotes and, per theme, the semiquantitative rating of interviewees mentioning it.

#### Intervention characteristics

HCPs found Participatient to be very user friendly. They reported that the app is easy to navigate and that its functionalities are clearly organized. The daily reminders for the catheter check and pain score were deemed beneficial for patients. However, many HCPs indicated that they already monitor pain scores and urinary catheter care.

HCPs suggested most patients prefer electronic communication over paper, making the app a valuable tool for sharing information. Most HCPs noted that while patients rarely discussed catheter use, the daily reminders could help prevent infections and could add usefulness to the app.

#### Inner setting

A facilitating factor mentioned by all the HCPs was their willingness to embrace healthcare innovations. However, many felt that the means and the amount of communication about Participatient were insufficient. While wards sent weekly newsletters to inform HCPs, not all the HCPs were reached, and patient engagement was often lacking. Promoting the app was frequently seen as an additional task not fitting with existing high workloads and as not worthwhile owing to the unknown benefit. This reluctance to promote was explained by HCPs partly because the app was not clearly integrated into their workflows and policies, which was seen as a barrier for large-scale endorsement of the app.

With respect to patient populations, several HCPs reported that the intervention was unsuitable for their ward. Patients often faced language barriers, felt too ill, had poor eyesight, or did not own a smartphone. Finally, the constantly changing composition of healthcare teams was reported as a hindering factor due to the departure of HCPs who had been trained for the mHealth intervention.

#### Outer setting

Some HCPs noted changes in the organizational structure of their hospitals, which caused disruptions that hindered the implementation of the app. One hospital’s policy was already committed to PE, which facilitated implementation.

#### Individual characteristics

A major facilitating factor was that most HCPs recognized numerous benefits in involving patients in their healthcare process and were open to increasing PE.

Overall, HCPs expressed a positive attitude toward the app’s information, indicating that providing medical information through Participatient is more reliable than patients conducting uneducated internet searches.

HCPs also mentioned that app usage is likely to be influenced by patients’ individual characteristics, such as their age or health status and whether they prefer to be involved or to have a more passive attitude regarding their health.

#### Process

One aspect of the implementation process that was seen as a major barrier was a lack of clarity regarding the project’s goal and the specific responsibilities and roles of HCPs in the process. For example, HCPs were unaware that promoting the app to patients was in part their responsibility. Nevertheless, some HCPs reported feeling highly involved in the implementation process by the project team, by participating in planning clinical lessons, kicking off, and becoming very important participants (VIPs). The clinical lessons and kick-off events were considered informative and interactive. However, the number of clinical lessons per ward was considered insufficient for wards with large staff. Additionally, the provided information on the app focused too narrowly on the catheter check and its medical background, rather than on the broader, other valuable content of the app.

HCPs suggested several follow-up events to improve their knowledge about Participatient and to garner more support. Additionally, HCPs recommended organizing meetings to discuss points of success and areas for improvement.

## Discussion

This study shows that patients find the evaluated app for PE in infection prevention very acceptable. Furthermore, after implementation of the Participatient app we noted a positive effect on PE through patients’ increased trust in physicians, although no further significant impact on perceived quality of care was observed.

Based on the TAM, users’ acceptance of the app was high, with mean scores of 4.1/5 for usefulness and 3.6/5 for ease of use, and most of them intended for future app use. These TAM scores are high compared to those reported in other studies.^22–25^ The high users’ acceptance rates of the app fulfill the precondition for implementation.

With 249 (7.6%) registered app users, the app utilization rate was low. This rate is in line with the utilization rate of 8.8% in our feasibility trial.^10,12^ Lack of smartphone ownership, privacy concerns and inability to download and install the app were named as primary barriers for app use.^10^

PE and quality of care at baseline were rated high and did not significantly change after implementation, except for patients’ trust in their physician which was higher after the implementation. This limited change could be due to the positive attitudes at baseline toward PE in the wards and to the reported good perceived quality of care of physicians, nurses, and overall. The increase in trust in their physician associated with the intervention may represent a causal effect. Trust can be gained by transparency, knowledge, mutual respect, the disclosure of mutual information, etc.^26,27^ The intervention’s main feature is stimulating information exchange and transparency of the treatment, which could have contributed to increased trust. Additionally, a trend toward more positive ratings of PE and quality of care was observed after the intervention. However, the associations and trends found were not controlled for possible confounders and were not validated in control groups; thus, causality is not proven.^28^

For the HCPs, the major barrier to implementation was the perceived lack of clear communication on the goals of the project with defined roles and involving the appropriate individuals. Damschroder *et al.* describe these barriers as part of the “Networks and communications” constructs of implementation.^20^ Clear communication on the goals, open feedback and review with the staff, and alignment of the goals all contribute to effective implementation. Additionally, effective implementation requires planning, engaging appropriate individuals, executing according to the plan, and reflecting with the team throughout the implementation process.^20^ In our study, the implementation plan set up by the project team focused on communication and engaging HCPs. However, despite their involvement and the communication on goals and roles, HCPs on several hospital wards did not promote the project among patients. Barriers in the same implementation domains “inner setting” and “process,” were described by Dekker *et al.* as main influencers of successful clinical implementation of infection prevention link nurse programs.^29^ Had there been more attention to assuring execution according to the plan and reflecting on the steps and results with the team, results of the implementation may have been different.

Although they were eligible and welcome, the wards’ physicians did not participate in the interviews on barriers and facilitators. That the physicians did not participate in the interviews is a potential barrier to PE implementation. Miller-Rosales *et al.* described modest adoption of PE strategies by U.S. physician practices.^30^ The extent to which PE occurs may depend on internal practice capabilities.

As a strategy to guide implementation, we identified VIPs among the HCPs during the clinical lessons was based on their enthusiasm, credibility, and clinical knowledge. These selection criteria are referred to in the literature as successful behavior characteristics of local champions that support implementation.^31,32^ Bunce *et al.* suggested that not only the strategy of local champions but also the appropriate operationalization (ie, the identification and preparation of champions) are necessary for effective practice change. However, notwithstanding the attention to identification and preparation of the VIPs, in interviews HCPs perceived the project lacked VIP involvement during the process. We recognize that a given VIP’s decision to participate in the implementation had triggered assumptions by our study team regarding organizational support for the targeted change, and VIP engagement in that change. However, these assumptions did not hold true in all VIPs, which appears to have influenced outcomes.

During the development of the intervention, we involved clinical patients and HCPs to adjust the app to the needs of the end users. However, the interviews revealed that the HCPs missed the compatibility of the intervention with the wards’ workflow. This project was initiated because the existing workflow lacked adherence to catheter and infection prevention protocols, which we sought to improve. The HCPs’ perceived influencing factors for a successful implementation were considered in this implementation. However, engaging HCPs in using new systems has been reported challenging.^33,34^

A major facilitator for implementation was the adaption of the app’s information to the ward and hospital. Another important facilitating factor was that HCPs in general were positive about engaging patients in their own healthcare process.

The study’s strengths lie in its scale and scope, covering 13 wards across four hospitals and analyzing a substantial sample of patient responses on the intervention’s acceptability and impact. The 22 healthcare professionals that were interviewed represented a diverse, though not exhaustive, subset. The inclusion of various ward types and medical specialties further enhances its comprehensiveness.

However, a limitation of this study is the selected sample, which included only patients who could complete a paper or digital questionnaire and certain healthcare professionals (nurses, senior nurses, and care managers). To provide a more comprehensive overview, future research should also consider the perspectives of other patients, hospital policymakers, and physicians.

The implementation strategy evaluated in the present study was designed to fulfill all the conditions considered crucial for implementation. Nevertheless, the level of adoption of the app remained low. HCPs still imputed their failure to promote the use of the app to insufficiencies in training and communication and to a misfit between the app and their existing workflow. The main findings are in line with previous findings on barriers and facilitators to implementation. However, assessing these influencers in the context of infection control with the concepts of patient engagement and mHealth is a novelty. The findings underscore the need to verify whether there may be additional, less evident barriers to the adoption of Participatient to engage patients in preventing CAUTIs specifically, and more in general, to the adoption of mHealth tools that support PE.

## Supporting information

Bentvelsen et al. supplementary material Bentvelsen et al. supplementary material

## Data Availability

The study protocol is made freely available online for researchers to access [DOI: 10.2196/28314]. Data collected during this study, including de-identified individual participant data and a data dictionary, will be made available to investigators. Data will be available beginning 3 months and ending 10 years following November 2023. Researchers must have their study protocol approved by an independent review committee identified for this purpose. Proposals should be directed to the chief investigator, Dr. Karin Ellen Veldkamp (k.e.veldkamp[at]lumc.nl); to gain access, data requestors will need to sign a data access agreement.

## Supplementary material

The supplementary material for this article can be found at https://doi.org/10.1017/ice.2025.10211
