# Supplementary material for: Evaluating the implementation of a patient engagement mHealth application in clinical infection prevention
Source: Infect Control Hosp Epidemiol. 2025 Aug 26;46(10):1020–6. doi: 10.1017/ice.2025.10211 (PMC12615119; doi:10.1017/ice.2025.10211)
Supplement: Bentvelsen et al. supplementary material [file S0899823X25102110sup001.docx]

**Evaluating the implementation of a patient engagement mHealth application in clinical infection prevention**

# Supplement

## Supplementary Table S1. Characteristics of the participants in the patient engagement and quality of care assessment before and after the implementation of the mHealth intervention

|  | Baseline | |  | Intervention | |  |
| --- | --- | --- | --- | --- | --- | --- |
|  | N=268 | Percentage |  | N=190 | Percentage |  |
| Gender |  |  |  |  |  |  |
| Male | 142 | 53.0 |  | 90 | 47.4 |  |
| Female | 126 | 47.0 |  | 100 | 52.6 |  |
| Age group |  |  |  |  |  |  |
| 18-29 | 25 | 9.3 |  | 12 | 6.3 |  |
| 30-39 | 27 | 10.1 |  | 13 | 6.8 |  |
| 40-49 | 25 | 9.3 |  | 12 | 6.3 |  |
| 50-59 | 43 | 16.0 |  | 38 | 20.0 |  |
| 60-69 | 70 | 26.1 |  | 65 | 34.2 |  |
| 70-79 | 45 | 19.6 |  | 35 | 18.4 |  |
| 80+ | 22 | 8.2 |  | 15 | 7.9 |  |
| Education level |  |  |  |  |  |  |
| Low | 92 | 34.3 |  | 86 | 45.3 |  |
| Middle | 91 | 34.0 |  | 43 | 22.6 |  |
| High | 82 | 30.6 |  | 54 | 28.4 |  |
| Other | 3 | 1.1 |  | 7 | 3.7 |  |
| Hospitals |  |  |  |  |  |  |
| AMC | 61 | 22.8 |  | 24 | 12.6 |  |
| HMC | 77 | 28.7 |  | 32 | 16.8 |  |
| LUMC | 106 | 39.6 |  | 98 | 51.6 |  |
| SG | 24 | 9.0 |  | 36 | 18.9 |  |

In total 483 questionnaires were collected, 458 of which were valid. The questionnaires taken verbally (n=7), with incomplete sociodemographics (n=17), or without an overall quality response (n=5) were excluded from the analyses.

AMC = Amsterdam University Medical Centre, HMC = Haaglanden Medical Centre, LUMC = Leiden University Medical Centre, SG = Spaarne Gasthuis.

Education levels according to the Dutch National Bureau for Statistics (CBS.nl): 1. Low: primary education, special needs primary education, prevocational secondary education (VMBO), secondary vocational education level 1 or equivalent (MBO 1), first three years of senior general secondary education (HAVO) or preuniversity secondary education (VWO). 2. Second: Senior years of senior general secondary education (HAVO) or preuniversity secondary education (VWO), secondary vocational education levels 2, 3 or 4 (MBO 2, 3 or 4). 3. High: higher vocational education (HBO), university bachelor’s or master’s degree, PhD.

## Supplementary Table S2. Characteristics of the interviewees for the implementation evaluation of the mHealth intervention

| *Respondent* | *Hospital - Ward* | *Gender* | *Position* |
| --- | --- | --- | --- |
| 1 | LUMC – TRAX | Female | Nurse |
| 2 | LUMC – TRAX | Female | Nurse |
| 3 | LUMC – VCH2 | Female | Care manager |
| 4 | LUMC – VCH2 | Female | Nurse |
| 5 | LUMC – KOHG | Female | Nurse |
| 6 | LUMC – KOHG | Female | Nurse |
| 7 | AMC – F6N | Female | Senior nurse |
| 8 | AMC – F6N | Female | Nurse |
| 9 | AMC – F6N | Female | Nurse |
| 10 | AMC – F7N | Female | Senior nurse |
| 11 | AMC – F7Z | Female | Senior nurse |
| 12 | HMC – H01A | Male | Senior nurse |
| 13 | HMC – H01A | Female | Nurse |
| 14 | HMC – H01A | Female | Nurse |
| 15 | HMC – H01B | Male | Nurse |
| 16 | HMC – C11 | Female | Care manager |
| 17 | HMC – C11 | Female | Senior nurse |
| 18 | HMC – C11 | Female | Senior nurse |
| 19 | Spaarne Gasthuis – Nephrology | Female | Senior nurse |
| 20 | Spaarne Gasthuis – Surgery | Female | Care manager |
| 21 | Spaarne Gasthuis – Surgery | Female | Nurse |
| 22 | Spaarne Gasthuis – Surgery | Female | Senior nurse |

## Supplementary Table S3. Questionnaire for patient engagement, quality of care, and acceptability assessment

Question 8 was added to the questionnaire in the version used after the implementation of the Participatient app.

Questionnaire on the quality of your hospital admission

The following questions are about your rating of the received care during your hospital admission. We would like to stress that all questions and responses are for research purposes only: the answers will not be shared with your nurses or physicians. Answers will be available only for the researchers of the Leiden University Medical Center (LUMC) and are anonymous, so not traceble to you. For contact details please see page 4.

*Prof. dr. Niels Chavannes, professor in primary care, LUMC*

*Drs. Robbert Bentvelsen, Clinical Microbiologist in training, LUMC*

*Dr. Rosalie van der Vaart, Assistant professor psychology, Leiden University*

*Rianne Beckerman, BSc psychology & Amber Gotz, BSc psychology, Leiden University*

1a. Sex:  Male  Female

1b. Age:  18-29 30-39 40-49 50-59 60-69  70-79 80+

1c. What is your highest level of completed education?

None / Primary school

Prevocational secondary education

Vocational secondary education

General secondary education

Higher vocational education or university degree

Other: ………..

2a. Do you have a peripheral intravenous catheter?  YES  NO.  I Don’t know

2b. Do you have a urinary catheter?  YES  NO.  I Don’t know

3. How do you rate the overall received care during your hospital admission?

Very poor Very good

1.  2.  3.  4.  5.  6.  7.  8.  9.  10.

4. How confident are you that you…

… know which questions to ask your physician?

not at all  a little confident  neutral  somewhat confident  very confident

… are able to get responses to all your questions from your physician?

not at all  a little confident  neutral  somewhat confident  very confident

… are able to optimally use the ward round (daily visit) of your physician?

not at all  a little confident  neutral  somewhat confident  very confident

… are able to express your main health problems to your physician?

not at all  a little confident  neutral  somewhat confident  very confident

… are able to get care for your main health problems from your physician?

not at all  a little confident  neutral  somewhat confident  very confident

5. Please note your agreement to the following statements. Tick one box per row.

|  | Completely disagree | disagree | agree | Completely agree |
| --- | --- | --- | --- | --- |
| I trust my physician completely |  |  |  |  |
| I think my physician is an expert |  |  |  |  |
| My physician takes good care of patients |  |  |  |  |

6. The physician(s) who takes care of me…

… knows my problems well

not at all.  a little.  mostly.  completely.

… lets me share the decision on the treatment or care I receive

not at all.  a little.  mostly.  completely.

… takes my opinion into account

not at all.  a little.  mostly.  completely.

… explains the treatment I receive

not at all.  a little.  mostly.  completely.

… cares for my wellbeing

not at all.  a little.  mostly.  completely.

… works well with the healthcare team

not at all.  a little.  mostly.  completely.

7. The nurse(s) who takes care of me…

… knows my problems well

not at all.  a little.  mostly.  completely.

… lets me share the decision on treatment or care I receive

not at all.  a little.  mostly.  completely.

… takes my opinion into account

not at all.  a little.  mostly.  completely.

… explains the treatment I receive

not at all.  a little.  mostly.  completely.

… cares for my wellbeing

not at all.  a little.  mostly.  completely.

… works well with the healthcare team

not at all.  a little.  mostly.  completely.

8. Your experience with the Participatient app

The following part of the questionnaire concerns your opinion of the Participatient app. This is an app which you can download for your phone or tablet during your hospital stay.

If you have not used the Participatient app yet, please skip this part of the questionnaire and proceed to the end of the form.

Which parts of the app have you used? More than one answer is allowed

Painscore.  Catheter check.  My ward.  More information.

The use of the app is easy

Completely disagree.  disagree.  agree.  Completely agree.

It is simple to find the information I am looking for in the app

Completely disagree.  disagree.  agree.  Completely agree.

The information in the app is easy to understand

Completely disagree.  disagree.  agree.  Completely agree.

The information in the app is useful

Completely disagree.  disagree.  agree.  Completely agree.

The app makes me feel more engaged in my care

Completely disagree.  disagree.  agree.  Completely agree.

I find it useful to get the advice to speak about care with my nurse/physician

Completely disagree.  disagree.  agree.  Completely agree.

During a next comparable hospital stay I would use the app again

Completely disagree.  disagree.  agree.  Completely agree.

What is your overall rating of the app?

Very poor Very good

1.  2.  3.  4.  5.  6.  7.  8.  9.  10.

What could be improved on the app?

|  |
| --- |

Thank you for completing this questionnaire. When you are finished, please put the form in the envelope, seal it, and ask your nurse to deliver it to the research team.

For information on the project, mail us at info[at]participatient.nl or call XXX.XXX.XXXX

## Supplement S4. Constructs for Assessment of Patient Engagement and Perceived Quality of Care

Patients’ ‘perceived efficacy in patient‒physician interactions’ was measured with the PEPPI-5 questionnaire. The PEPPI-5 contains five items that should be answered via a scale ranging from 1 to 5, with 1 meaning ‘Not at all confident’ and 5 meaning ‘Very confident’. An example question is ‘How confident are you in your ability to know what questions to ask your doctor?’. The total scores are summed, ranging from 5 (least self-efficacy) to 25 (most self-efficacy). Higher scores correlate with higher perceived efficacy in expressing their concerns. The PEPPI-5 is a validated assessment instrument, and the Dutch version has a Cronbach’s alpha of 0.92. [a,b]

Trust in physicians was measured with items from the Trust In Physicians short form (TRIP_sf). TRIP_sf consists of three items in which statements should be rated on a scale from 1 to 4, with 1 meaning ‘Do not agree at all’ and 4 meaning ‘Completely agree’. An example statement is ‘I trust my doctor completely’. The total score is summed and divided by three. The higher the final score is, the more trust in the physician is perceived by the patient. TRIP_sf is a validated assessment instrument with a Cronbach’s alpha of 0.93. [c]

The quality of the care providers was measured with six items based on the Quality of Care through the Patient's Eyes (QUOTE) questionnaire. This scale was used twice, once to measure patient satisfaction with the involvement of the physician and once to measure patient satisfaction with the involvement of the nurse. The items are phrased as statements, and patients rate each statement on a scale from 1 to 4, with 1 meaning ‘Not at all’ and 4 meaning ‘Definitely’. An example statement is “My nurse knows my problems well”. The means of the totals ranged between 6 (no perceived involvement) and 24 (most perceived involvement). The six items of the QUOTE have been validated in other studies, with two Cronbach's alphas of 0.83 and 0.87. [d,e]

Perceived quality of care overall was measured through a single question: “How do you feel about the quality of care overall in this hospital?”. The patients had to score their perceived quality of care on a scale from 1 to 10. A score of 1 indicates that the perceived quality of care is 'very bad', and a score of 10 indicates that the perceived quality of care is 'very good'.

References

1. Maly RC, Frank JC, Marshall GN, DiMatteo MR, Reuben DB. Perceived efficacy in patient-physician interactions (PEPPI): validation of an instrument in older persons. J Am Geriatr Soc. 1998 Jul;46(7):889-94. doi: 10.1111/j.1532-5415.1998.tb02725.x. PMID: 9670878.
2. Ten Klooster PM, Oostveen JC, Zandbelt LC, Taal E, Drossaert CH, Harmsen EJ, van de Laar MA. Further validation of the 5-item Perceived Efficacy in Patient-Physician Interactions (PEPPI-5) scale in patients with osteoarthritis. Patient Educ Couns. 2012 Apr;87(1):125-30. doi: 10.1016/j.pec.2011.07.017. Epub 2011 Sep 1. PMID: 21889864.
3. Ommen O, Janssen C, Neugebauer E, Bouillon B, Rehm K, Rangger C, Erli HJ, Pfaff H. Trust, social support and patient type--associations between patients perceived trust, supportive communication and patients preferences in regard to paternalism, clarification and participation of severely injured patients. Patient Educ Couns. 2008 Nov;73(2):196-204. doi: 10.1016/j.pec.2008.03.016. PMID: 18450408.
4. Van Campen C, Sixma HJ, Kerssens JJ, Peters L, Rasker JJ. Assessing patients' priorities and perceptions of the quality of health care: the development of the QUOTE-Rheumatic-Patients instrument. Br J Rheumatol. 1998 Apr;37(4):362-8. doi: 10.1093/rheumatology/37.4.362. PMID: 9619883.
5. Van der Vaart R, Drossaert CH, Taal E, Drossaers-Bakker KW, Vonkeman HE, van de Laar MA. Impact of patient-accessible electronic medical records in rheumatology: use, satisfaction and effects on empowerment among patients. BMC Musculoskelet Disord. 2014 Mar 26;15:102. doi: 10.1186/1471-2474-15-102. PMID: 24673997; PMCID: PMC3986693.

## Supplement S5. Procedure for the assessment of implementation barriers and facilitators in HCP interviews

After a month, the implementation process should be completed, and the novelty of the innovation should have decreased sufficiently to obtain a reliable assessment of HCPs’ experiences. The interviews were recorded and transcribed verbatim.

The interview started with an explanation of the aim of the interview and assurances regaring the anonymization and confidentiality of the data. The interviewer asked the participants to be as open and honest as possible. The interview scheme was based on the Consolidated Framework for Implementation Research and addressed the five domains of the framework (the intervention characteristics, the inner setting and the outer setting of a healthcare institution, the characteristics of individuals and the implementation process). The first question was an open-ended question regarding the experiences of the HCP with the mHealth application in general. The participants could reflect on any part of the application and its implementation that they found worthwhile to mention. On the basis of what the participant mentioned, the interviewer asked probing questions. During each interview, each of the five domains of the CFIR was addressed with follow-up open questions. For example, “Could you tell me something about your experiences with the app, for example, about its usability and its content?” and “Could you talk about how the setting in which you work affected the implementation of the intervention?”. If needed, further questions were asked in each domain to gain as much insight as possible into implementation barriers and facilitators and suggestions for future improvement of the intervention.

The HCP interviews lasted approximately 30 minutes. Responses concerning barriers and facilitators were coded and categorized via deduction per the CFIR domain. Responses were grouped in a coding scheme by two researchers, and themes were identified via induction. The three steps could be derived from open coding, the first stage in grounded theory methodology [f]. This is an approach whereby repeatedly reviewing and refining the coding of the interviews results in giving an organized meaning to the phenomena mentioned by HCPs in the interviews.

Reference:

1. Strauss A, Corbin J. (1998) Basics of qualitative research: Techniques and procedures for developing grounded theory. Thousand Oaks, CA: Sage Publications, Inc.
